# Valentino Syndrome With Histologically Confirmed Acute Appendicitis: A Rare Dual Pathology

**DOI:** 10.7759/cureus.115474

**Published:** 2026-08-30

**Authors:** Imran M Adam, Ahmed R Zubi, Ja Mann, Reema Mujthaba, Aminath Shaamath Naseer

**Affiliations:** 1 Department of General Surgery, Indira Gandhi Memorial Hospital, Male, MDV

**Keywords:** acute appendicitis, case report, dual pathology, perforated peptic ulcer, rare clinical presentation, valentino’s syndrome

## Abstract

Acute abdomen is a common surgical emergency with multiple etiologies; acute appendicitis and perforated peptic ulcer (PPU) are among the common. Valentino’s syndrome is a rare condition in which patients with PPU present with clinical features of acute appendicitis due to the flow of gastric contents along the right paracolic gutter, causing inflammation of the appendix. Diagnosis is often delayed and made during surgery.

We discuss a case of a 25-year-old male who presented with acute pain in the right lower quadrant. Initial assessment pointed toward acute appendicitis; however, on further evaluation, the patient was found to have a perforated prepyloric ulcer with 500 mL of seropurulent peritoneal fluid and an inflamed appendix. He underwent Graham’s patch repair of the prepyloric ulcer along with peritoneal lavage and appendectomy. Histopathology of the appendix revealed features suggestive of acute appendicitis, and biopsy from the prepyloric ulcer revealed an active ulcer with few *Helicobacter ​​​​​pylori*-like organisms, hence supporting the diagnosis of *H. pylori*-associated peptic ulcer. Post-operatively, the patient was initiated on *H. pylori *eradication therapy, and his recovery was uneventful. This case highlights the importance of clinical assessment and remaining alert for other possible etiologies of right lower quadrant pain in atypical presentations. Valentino’s syndrome, although rare, must still be considered in such cases of right lower quadrant pain with discrepancies in initial investigations and clinical findings. Often, Valentino’s syndrome is treated as acute appendicitis; however, prompt re-assessment and in-depth evaluation of the patient followed by timely surgical intervention can lead to optimal outcomes.

## Introduction

Acute abdomen is a clinical syndrome with multiple etiologies, many of which require emergency surgical intervention. Among these, acute appendicitis and perforated peptic ulcer (PPU) are common and potentially serious conditions. Acute appendicitis predominantly affects adolescents and young adults, whereas PPU is more frequently encountered in adults with risk factors such as smoking, chronic non-steroidal anti-inflammatory drug use, and *Helicobacter pylori* infection [1,2].

Valentino’s syndrome is a rare clinical entity in which a PPU mimics acute appendicitis due to the tracking of gastroduodenal contents along the right paracolic gutter, resulting in localized peritonitis in the right lower quadrant [3]. Its true incidence and prevalence remain unknown, with the available evidence derived predominantly from isolated case reports, small case series, and literature reviews, precluding reliable population-level epidemiological estimates [4]. Surgical exploration may reveal a mildly inflamed or normal appendix, potentially leading to misdiagnosis and failure to identify the underlying gastroduodenal perforation [5].

The coexistence of a PPU with histologically confirmed acute appendicitis is exceptionally rare and poses an additional diagnostic challenge. Identification of one pathology may result in the second being overlooked [6]. Therefore, a high index of suspicion and thorough intraoperative evaluation are important, particularly when clinical, radiological, and operative findings are discordant.

The diagnostic workup of patients presenting with an acute abdomen is critical. In suspected PPU, routine laboratory investigations are often non-specific, while computed tomography (CT) is the preferred imaging modality because it can identify pneumoperitoneum, localize the site of perforation, and demonstrate associated intra-abdominal findings. Where CT is unavailable, plain radiography and water-soluble contrast studies may assist in establishing the diagnosis [7]. Similarly, CT has high diagnostic accuracy for acute appendicitis and is particularly useful when the clinical presentation or ultrasonographic findings are equivocal [8].

This case describes a patient with Valentino’s syndrome due to a perforated prepyloric ulcer associated with histologically confirmed acute appendicitis, highlighting the diagnostic challenges posed by overlapping intra-abdominal pathologies and the importance of reassessment when clinical and radiological findings are discordant. This case report has been reported in line with the SCARE (Surgical CAse REport) guidelines [9].

## Case presentation

A 25-year-old Nepali male smoker with no significant past medical or surgical history presented to the emergency department with a one-day history of right lower abdominal pain. The pain was described as non-radiating and was associated with loss of appetite. However, there was no associated fever, nausea, or vomiting. On arrival, his vital signs were as follows: pulse rate, 100 beats per minute, regular with good volume; blood pressure, 112/70 mmHg; oxygen saturation, 98% on room air; respiratory rate, 30 breaths per minute; and temperature, 37.2°C.

Biochemical investigations were within normal limits apart from an elevated INR, which was considered most likely to be a laboratory error (Table 1). An initial erect abdominal X-ray obtained 16 minutes after presentation demonstrated a ground-glass appearance with no evidence of pneumoperitoneum. Fecal loading was also visualized in the ascending, transverse, and descending colon.

**Table 1:** Laboratory investigations

Parameter	Result	Unit/Remarks	Reference range
Total leukocyte count (TLC)	9.66	×10³/µL	3.99-8.71 × 10³/µL
Neutrophils/lymphocytes	50.9/37.6	%	17.5%-69.5%
C-reactive protein (CRP)	<0.10	mg/dL	0-0.5 mg/dL
Hemoglobin (Hb)	14.4	g/dL	11.9-15.4 g/dL
Packed cell volume (PCV)	41.6	%	36.2%-46.3%
Platelet count (PLT)	295	×10³/µL	151-304 × 10³/µL
Total bilirubin	1.6	mg/dL	0.2-1.2 mg/dL
Direct bilirubin	0.2	mg/dL	0.0-0.5 mg/dL
AST (SGOT)	47	U/L	5-34 U/L
ALT (SGPT)	41	U/L	0-55 U/L
Serum amylase	56	U/L	23-85 U/L
Serum lipase	15.72	U/L	0-160 U/L
Serum creatinine	0.85	mg/dL	0.72-1.25 mg/dL
Sodium (Na⁺)	138	mmol/L	136-145 mmol/L
Potassium (K⁺)	4.4	mmol/L	3.5-5.1 mol/L
INR	3.21	-	0.8-1.2
Repeat INR	1.59	-	0.8-1.2
Random blood sugar (RBS)	90	mg/dL	60-11 mg/dL

Whole-abdomen ultrasonography (USG) performed six hours after presentation revealed a small amount of free fluid in the right iliac fossa, with probe tenderness in the right iliac fossa (Figure 1, Panels A and B). The appendix was not visualized. Subsequently, a surgical consultation was obtained within six hours of arrival at the emergency department.

**Figure 1:**
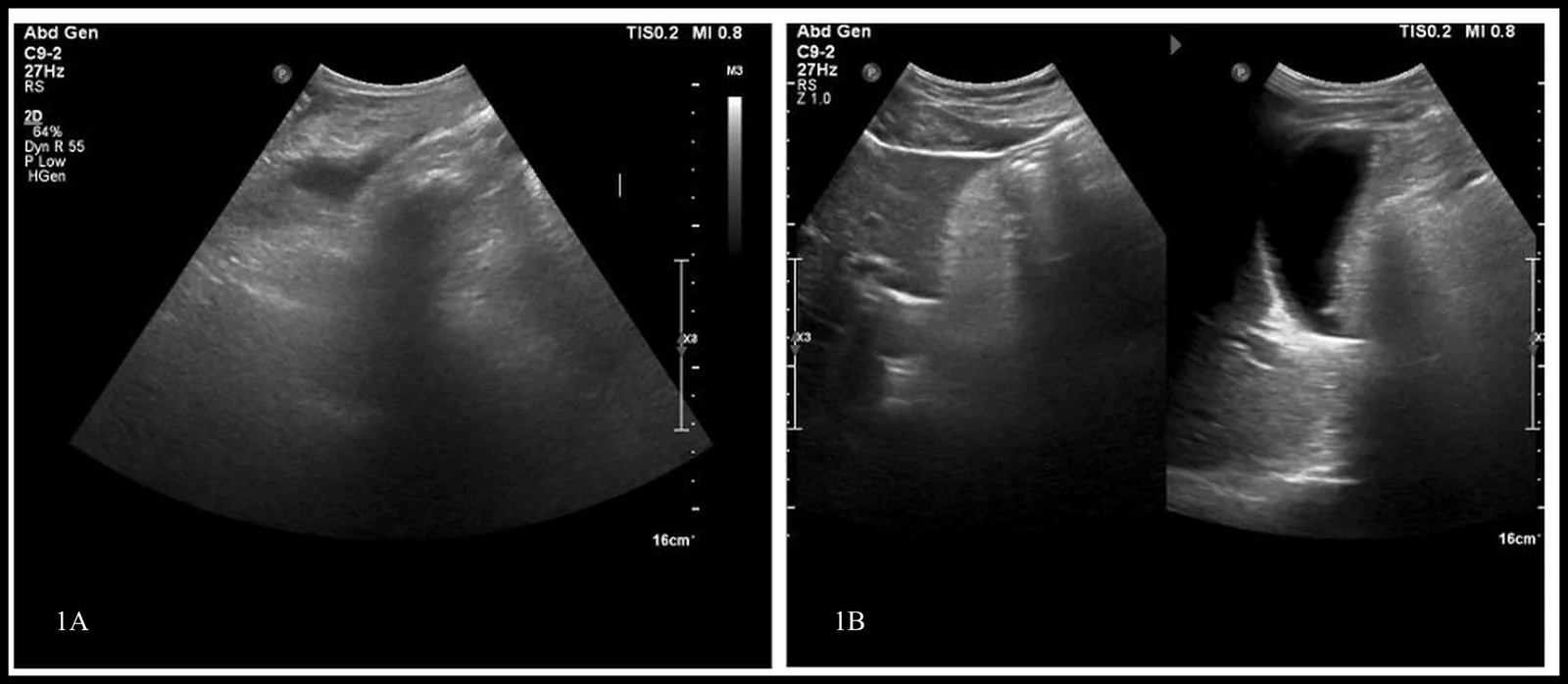
Whole-abdomen ultrasonography. (A) Mild free fluid in the right iliac fossa. (B) No fluid in the hepatorenal pouch.

During evaluation by the surgical team, abdominal examination revealed tenderness over the umbilical, right and left iliac fossa, and hypogastric regions, with generalized abdominal guarding. Rebound tenderness was most marked in the right iliac fossa, along with a positive Rovsing’s sign. However, there was no abdominal wall rigidity, and obturator, psoas, and Dunphy’s signs were absent. The inguinal hernial orifices were normal, with no visible or palpable hernia. There was no umbilical hernia. Renal angle tenderness was absent.

The calculated Alvarado score was 4, suggesting a low probability of appendicitis [9]. Based on the clinical presentation and USG findings, a provisional diagnosis of acute appendicitis was made. However, given that the patient was hemodynamically stable and had a low Alvarado score, contrast-enhanced CT of the abdomen was performed.

CT of the abdomen showed an inflamed and dilated appendix with enhancing walls, with a maximum diameter of 8.7 mm, suggestive of acute appendicitis (Figure 2, Panel A). Adjacent fat stranding was seen, with mild peri-appendiceal fluid and fluid in the right perinephric region.

**Figure 2:**
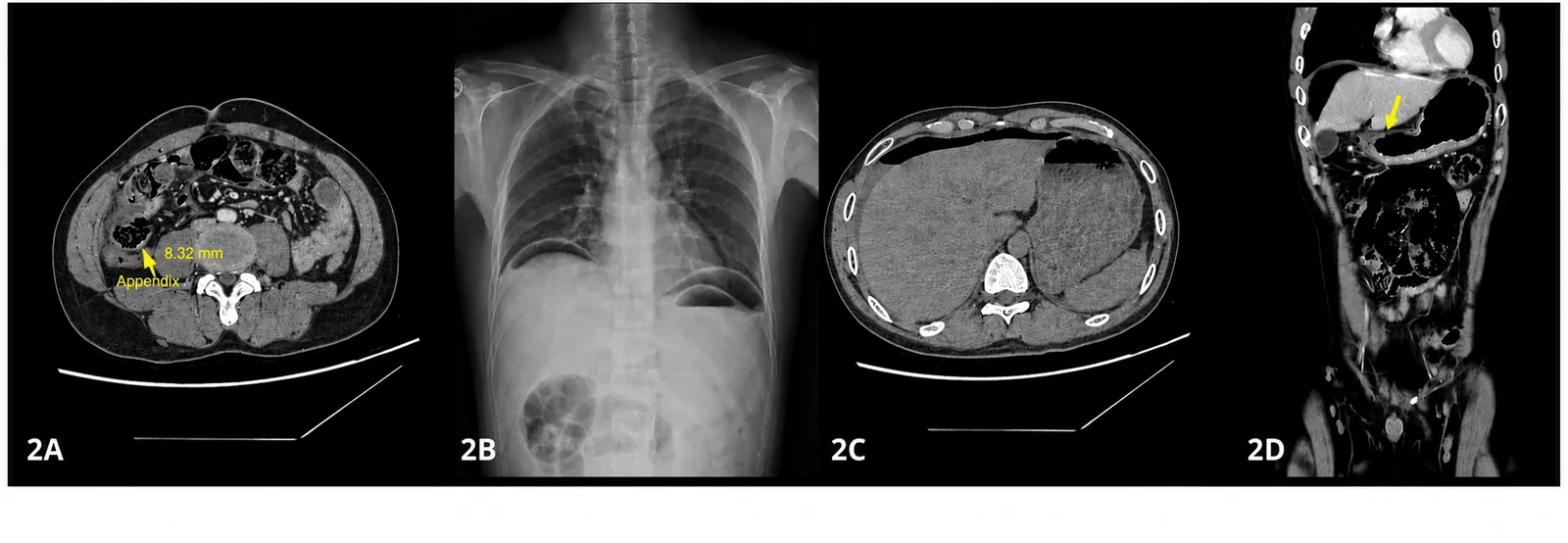
(A) CT scan demonstrating a dilated appendix (8.3 mm). (B) Erect chest radiograph showing free subdiaphragmatic air consistent with pneumoperitoneum. (C) CT imaging demonstrating pneumoperitoneum and suspected site of perforation. (D) Coronal CT showing the pneumoperitoneum.

Accordingly, the patient was scheduled to undergo emergency laparoscopic appendectomy. Prior to surgery, a preoperative erect chest X-ray obtained 14 hours after presentation revealed pneumoperitoneum (Figure 2, Panel B). The CT images were therefore reviewed, which confirmed the presence of pneumoperitoneum (Figure 2, Panels C and D), with fluid in the right subhepatic region. The CT report was subsequently revised.

The patient was reassessed 18 hours after the initial assessment and was found to have involuntary guarding in all quadrants. Rebound tenderness was present, along with obliteration of liver dullness. These findings raised suspicion of peritonitis secondary to hollow-viscus perforation rather than acute appendicitis alone. Intravenous fluid resuscitation was continued, and broad-spectrum antibiotic therapy with ceftriaxone 1 g was initiated.

Written informed consent was obtained, and the patient was taken for emergency diagnostic laparoscopy. Intraoperatively, 1 unit of fresh frozen plasma (FFP) was transfused because of the deranged coagulogram.

Laparoscopy revealed approximately 500 mL of seropurulent peritoneal fluid, with adhesions between the abdominal wall, omentum, liver, and stomach (Figure 3, Panels A and B). Peritoneal fluid was sent for culture and sensitivity and cytological examination.

**Figure 3:**
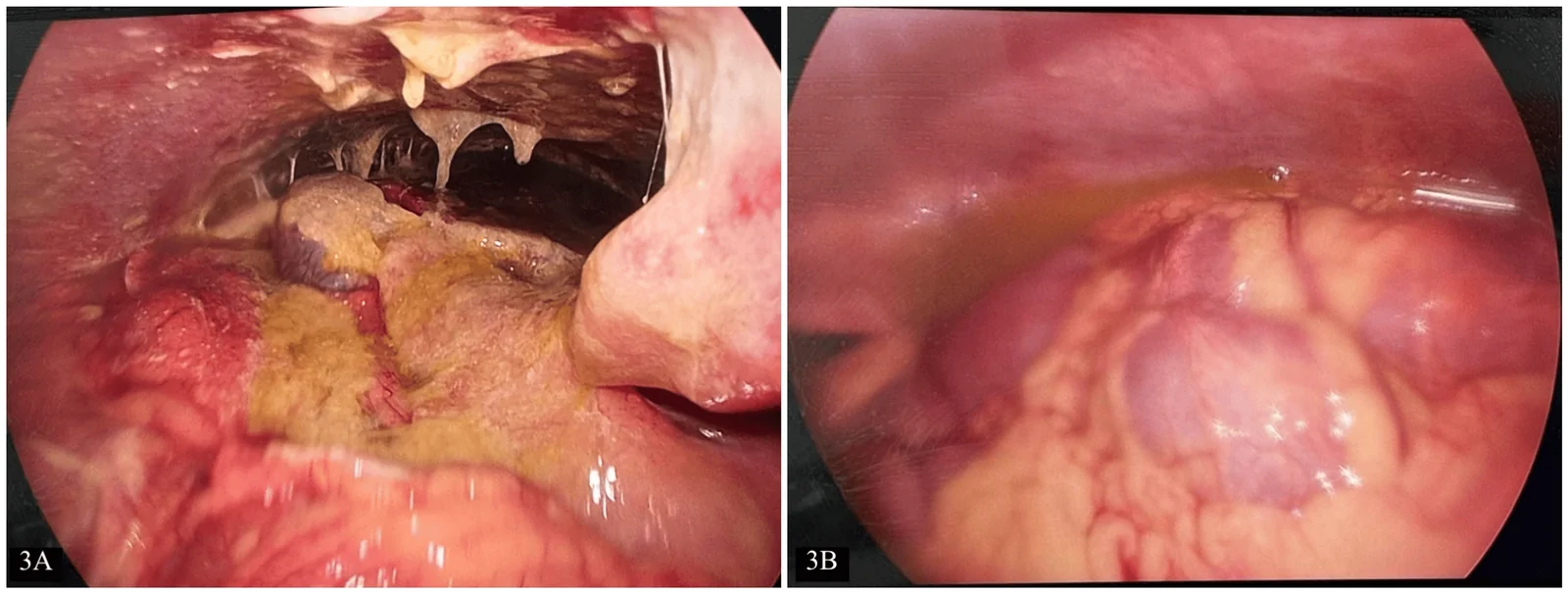
Laparoscopic views showing adhesions and intra-peritoneal fluid collection: (A) hepatic flexure and (B) right iliac fossa

Due to technical difficulties, the exploratory laparoscopy was converted to laparotomy. The peritoneal cavity was irrigated with copious amounts of normal saline. Intraoperatively, a perforated anterior prepyloric ulcer measuring 3 x 3 mm with induration at the ulcer edge was identified (Figure 4, Panel A).

**Figure 4:**
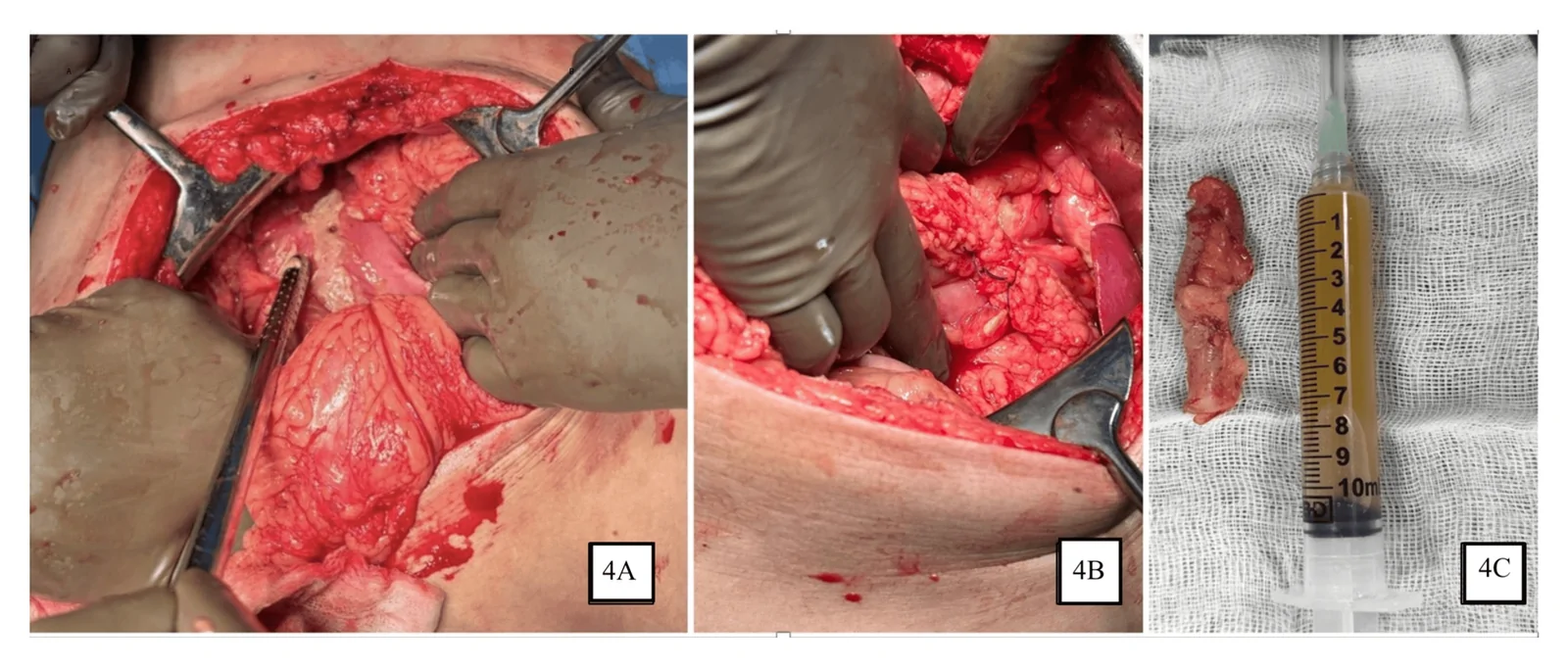
Intra-operative findings: (A) perforated prepyloric ulcer, (B) Graham’s patch repair, and (C) gross specimen of the resected appendix alongside aspirated intraperitoneal purulent fluid

Additionally, the appendix, in a paracaecal position, was minimally inflamed, with pus flakes around it. The base of the appendix was healthy. An open appendectomy was performed. The bowel was examined from the duodenojejunal junction to the ileocecal junction. The ileum adjacent to the appendix was mildly inflamed. Otherwise, no other perforations or pathological findings were identified.

Peritoneal lavage and Graham’s patch repair of the anterior prepyloric ulcer were therefore performed (Figure 4, Panel B). A biopsy was taken from the prepyloric ulcer for histopathological examination. A 20-Fr subhepatic drain was placed.

Postoperatively, the patient was shifted to the high dependency unit for close monitoring and was treated with intravenous antibiotics (ceftriaxone for six days and metronidazole for three days), fluids, and analgesics; nonsteroidal anti-inflammatory drugs were avoided. The fluid culture and sensitivity report revealed no bacterial growth, and cytological examination of the fluid showed inflammatory changes without malignant cells. The patient's clinical condition improved, and he was started on feeds and mobilized. The nasogastric tube was removed on postoperative day 5, and the subhepatic drain was removed on postoperative day 6.

Prior to discharge, the patient received oral triple eradication therapy for *H. pylori* with amoxicillin (1 g twice daily), metronidazole (400 mg twice daily), and pantoprazole (40 mg twice daily). He was discharged in a hemodynamically stable condition on postoperative day 7.

During follow-up visits, the patient recovered well without complications. Histopathological examination of the ulcer tissue confirmed active ulceration with *H. pylori*-like organisms. *H. pylori *testing could not be performed because the service was temporarily unavailable. Furthermore, biopsy of the appendix confirmed acute appendicitis. Cytological examination of the peritoneal fluid showed inflammation but no malignant cells.

## Discussion

Valentino’s syndrome is a rare condition in which patients with a PPU present with clinical features mimicking acute appendicitis due to spillage of gastric or duodenal contents tracking along the right paracolic gutter into the right iliac fossa, resulting in localized chemical peritonitis. In this unusual presentation, lower abdominal pain may precede and obscure the symptoms of PPU, often leading to delayed or missed diagnosis, with the definitive diagnosis frequently made intraoperatively.

Unlike classical Valentino’s syndrome, in which the appendix is often normal or minimally inflamed, our case demonstrated histologically confirmed acute appendicitis coexisting with a perforated prepyloric ulcer, representing an extremely rare dual pathology.

Acute appendicitis is one of the most common surgical emergencies worldwide, with a global age-standardized incidence rate of 214 per 100,000 population in 2021 [10]. Peptic ulcer disease is also common, with a lifetime prevalence of 5%-10% and an annual incidence of 0.1%-0.3% [6]. South Asia has the highest reported age-standardized prevalence rate [11]. Although perforation is uncommon, it remains the most frequent indication for emergency surgery among ulcer-related complications and accounts for approximately 40% of ulcer-related deaths [7].

Only a few cases of Valentino’s syndrome with associated appendiceal inflammation have been reported in the literature [12-14]. These reports demonstrate the considerable diagnostic overlap between PPU and acute appendicitis, with the underlying gastroduodenal perforation potentially being identified only after further imaging or surgical exploration [12-14]. This emphasizes the importance of considering an alternative or additional intra-abdominal pathology when findings are not fully explained by the initial diagnosis.

Normal inflammatory markers do not exclude appendicitis, as 2%-8.5% of patients with histologically confirmed appendicitis may have normal white blood cell counts and CRP levels [15]. Peptic ulcer perforation accounts for approximately 70% of pneumoperitoneum cases, whereas perforated appendicitis accounts for 0%-7% of cases [16]. Therefore, pneumoperitoneum in a patient with suspected appendicitis should prompt evaluation for an alternative source of hollow-viscus perforation.

CT is the diagnostic modality of choice for Valentino’s syndrome, with reported sensitivity exceeding 90%. CT was performed in 41%-70% of reported cases [5,17]. Surgical management involves repair of the PPU with or without appendectomy. Open repair remains common despite the safety of laparoscopic approaches [5,18].

Routine appendectomy in Valentino’s syndrome remains controversial. Appendectomy is recommended when the appendix appears inflamed, whereas removal of a normal appendix remains debatable [19]. In our case, appendectomy was justified by the presence of gross inflammation.

## Conclusions

Perforated prepyloric ulcers can present with signs of appendicitis, a presentation well recognized as Valentino’s syndrome. This rare syndrome may be mistaken for and treated as acute appendicitis, with the diagnosis often being made when a PPU is discovered intraoperatively. Therefore, careful abdominal evaluation and timely surgical intervention are essential to achieve optimal outcomes.

Key lessons include maintaining a broad differential diagnosis, reassessing the patient when the clinical picture changes, and carefully reviewing imaging findings. Prompt surgical intervention, with the flexibility to escalate from laparoscopy to laparotomy, remains the cornerstone of management. With timely intervention, even complex dual pathologies can have favorable outcomes.
